# Enrichment of the rebaudioside A concentration in *Stevia rebaudiana* extract with cyclodextrin glycosyltransferase from *Bacillus licheniformis* DSM 13

**DOI:** 10.1002/elsc.202100111

**Published:** 2021-11-10

**Authors:** Réka Czinkóczky, Áron Németh

**Affiliations:** ^1^ Department of Applied Biotechnology and Food Sciences Budapest University of Technology and Economics Budapest Hungary

**Keywords:** *Bacillus licheniformis*, bioconversion, CGTase, rebaudioside A, *Stevia rebaudiana*

## Abstract

*Stevia rebaudiana* is a sweet herbaceous perennial plant, which is frequently used in the preparation of plant‐based sweeteners. The demand for such sweeteners continues to increase due to purposeful nutrition and modern‐day metabolic syndromes. More than 20 types of steviol glycosides provide a sweet taste, which are more than 300 times sweeter than sucrose. They are formed of two main components, namely stevioside and rebaudioside A. Only a handful of studies have dealt with *Stevia rebaudiana* leaf extracts, the conversion of pure stevioside into the preferred rebaudioside A is more common. The aim of this study was to enrich the rebaudioside A content of *Stevia rebaudiana* leaf extract using enzymatic bioconversion by applying fermented cyclodextrin glycosyltransferase from *Bacillus licheniformis* DSM13. Two differently processed plant materials, namely dried and lyophilized *Stevia rebaudiana* plants, were extracted and compared. Following the bioconversion, the rebaudioside A content was on average doubled. The maximum increase was fivefold with a 70–80% conversion of the stevioside.

**Abbreviations**
CGTase: cyclodextrin glycosyltransferase
HPLC: high‐performance liquid chromatography
Reb A: rebaudioside A
RSE: relative standard error

## 1 INTRODUCTION

Nowadays, the demand for natural, low‐calorie sweeteners which can be consumed by those with metabolic disorders (e.g. cardiovascular disease, diabetes and obesity) as a substitute for table sugar is increasing. *Stevia rebaudiana* (Bertoni) is the perfect candidate to meet the aforementioned demand, since it contains calorie‐free sweet molecules called steviol glycosides. *Stevia rebaudiana* is a sweet perennial herb that originates from Paraguay and Brazil. Its sweet taste is caused by steviol glycosides which are mostly located in its leaves. The two main sweetening components, that are, stevioside and rebaudioside A (reb A), are contained in its leaves and amount to 4–13% and 2–4% of their dry matter, respectively. Apart from *S. rebaudiana*, other species are also potential sweeteners, e.g. *S. phlebophylla, S. anisostemma, S. bertholdii, S. crenata, S. eupatoria, S. lemmonii, S. micrantha, S. plummerae, S. salicifolia, S. serrata and S. viscida*. However, among them, *S. rebaudiana* exhibits the highest level of sweetness, its steviol glycosides are more than 300 times sweeter than sugar (sucrose) [1, 2, 3].

**PRACTICAL APPLICATION**

We used the fermented cyclodextrin‐glycosyltransferase (CGTase) enzyme of *Bacillus licheniformis* DSM13 to investigate its applicability on the conversion of stevioside into rebaudioside A, resulting more beneficial plant extract. Our study revealed that long term stored *Stevia rebaudiana* biomass is also suitable for recovering stevioside, and this raw extract is well convertible into rebaudioside A. Most of the recent studies report only the enzymatic bioconversion of pure stevioside but for industrial purposes less expensive raw extracts are more feasible.

**FIGURE 1:**
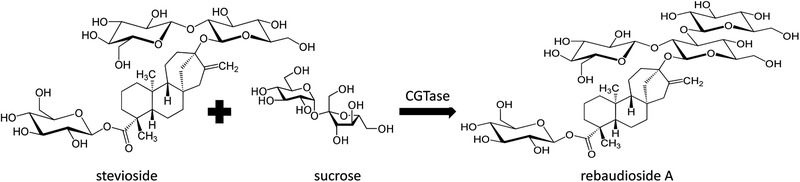
Bioconversion of stevioside to rebaudioside A using sucrose as a glycosyl donor and CGTase as a catalyst

*Stevia rebaudiana* contains various types of glycosides. Although the most abundant sweet terpenoid component is stevioside, it has a bitter aftertaste. The second most copious sweet molecule is rebaudioside A, which is sweeter and does not cause this unpleasant aftertaste. Therefore, the enzymatic bioconversion of stevioside to rebaudioside A improves the quality and properties of this sweet product [4]. Rebaudioside C, D, E and F, dulcoside A as well as steviolbioside are minor sweet terpenoid compounds in this plant [5]. The commercial production of stevioside began in the late 1970s [6]. Due to its bitter aftertaste, its use in human nutrition is restricted [7]. Chemical and enzymatic modifications are improving the taste and solubility of this product. The difference between the composition of stevioside and rebaudioside A is only one glucose unit (Figure 1). Different enzymatic systems were studied and summarized by Yücesan and Altuğ (2020). For the enzymatic modification of steviol glycosides, cyclodextrin glycosyltransferases (CGTase), α‐ and β‐glucosidases as well as galactosidase systems, and the bioconversion systems of UDP‐glycosyltransferases are capable of reducing the unpleasant aftertaste or enhancing their level of sweetness [8]. The published CGTase catalyzed bioconversions in the field of stevioside conversion are summarized in Table 1. From Table 1, it can be seen that stevioside is applied more frequently than *S. rebaudiana* leaf extract. Apart from the conventional heated and stirred long reaction times, in some cases, exceptionally short reaction times in microwave reactors yield higher conversion percentages. By analyzing recent studies, the average conversion percentage of stevioside is 71%; however, these values refer to chemically pure stevioside both in microwave‐assisted and conventional reactions. Apart from CGTase, β‐1,3‐glucanase can also be applied in these types of bioconversions. Singla and Jaitak (2016) tested the effect of β‐1,3‐glucanase extracted from *Irpex lacteus* on curdlan as a glycosyl donor. The stevioside conversion percentage was 62.5%, which is quite remarkable given that traditional methods of bioconversion were applied to produce rebaudioside A [9]. With the help of UDP‐glycosyltransferase 76G1 and the co‐expression of sucrose synthase in *Pichia pastoris* GS115, Chen et al. reached a 88.9% stevioside conversion to produce 261.2 mM rebadudioside A in a newly implemented feeding cascade bioconversion [10]. Both conventional and microwave‐assisted bioconversions have advantages and disadvantages. Although the short reaction time has a beneficial effect on the batch time, the main advantage of conventional enzymatic bioconversion is that no special reactors are required.

**TABLE 1:** Summary of the enzymatic modification of steviol glycosides using cyclodextrin glycosyltransferase systems

Enzyme source	Enzyme activity	Glycosyl donor	Glycosyl acceptor	pH [‐]	T [°C]	Reaction time	Reaction type	Stevioside conversion [%]	Reb A conversion [%]	Stevioside content [%]	Reb A content [%]	Reference
*Trichoderma viridae* cellulase Onozuka R‐10	N/D	Soluble starch	Stevia leaf	4.6	50	10 h	Conventional	N/D	N/D	14.40	66.00	[26]
		Sucrose								44.36	6.65	
		Lactose								48.01	6.48	
		Glucose								60.00	7.00	
		Β‐cyclodextrin								24.58	38.26	
*Bacillus firmus* β‐CGTase	0.12‐4.00 U/g	Β‐cyclodextrin	Stevioside	1‐11 (7)	10‐80 (50)	1 min	Microwave reactor 80 W	70.00	N/D	N/D	N/D	[24]
*Thermoanaerobacter* Toruzyme 3.0 L CGTase	370 U/mL	Gelatinized corn starch	Stevioside	6‐8	60	3 min	Microwave reactor 50 W	61.20	N/D	N/D	N/D	[25]
*Bacillus sp*. BL‐12 β‐CGTase	205 U/mg	Maltodextrin	20 g/L stevioside	8.5	40	12 h	Conventional	76.00	N/D	N/D	N/D	[27]
*Thermoanaerobacter* Toruzyme 3.0 L CGTase	10 U/g stevioside	Corn starch	stevioside	5‐6	60	3 h	Conventional	77.11	N/D	N/D	N/D	[28]
UDP‐glycosyltransferase UGT76G1 in *Pichia pastoris* GS115 and sucrose synthase from *Vigna radiata*	About 180 U/g/(cell dry weight)	800 mM sucrose	160 mM stevisodie	7	50	26 h	Fed‐batch cascade bioconversion	88.9	N/D	N/D	N/D	[10]

*Bacilli* are well‐known microorganisms, various strains of which are used in industrial applications. They are capable of producing different enzymes, including cyclodextrin glycosyltransferase [11, 12]. CGTase can catalyze various reactions, including cyclization, coupling, disproportionation and hydrolysis [13]. Apart from the modification of steviol glycosides, CGTase can be applied in the synthesis of naringin and neohesperidin glycosides [14]. CGTase enzymes from *Bacillus stearothermophilus* B‐5076 and *Bacillus macerans* BIO‐4 m were able to perform efficient enzymatic modifications of stevioside, as well rebaudioside A. The formed glycosylated derivatives successfully separated by HPLC method. These enzymatic bioconversions were improved the gustatory properties of the steviol glycosides [15].

In this study, the enrichment of the rebaudioside A content in the *S. rebaudiana* leaf extract by the transglycosylation of steviol glycosides using cyclodextrin glycosyltransferase extracted from *Bacillus licheniformis* DSM13 is presented. In our experiments, two differently processed ‐ heat‐dried and lyophilized ‐ plant biomasses were compared to determine how the pretreatment of biomass from *S. rebaudiana* plants influences the transglycosylation of stevioside to rebaudioside A.

## 2 MATERIALS AND METHODS

### 2.1 Microbial strain

*Bacillus licheniformis* B.01470 (DSM 13) was purchased from the National Collection of Agricultural and Industrial Microorganisms in Hungary.

### 2.2 Cyclodextrin glycosyltransferase fermentation

The enzyme was produced in the Horikoshi II medium. All chemicals were purchased from Reanal Laboratory Chemicals Ltd., Hungary. The Horikoshi II medium contained 1.0% soluble starch, 0.5% peptone, 0.5% yeast extract, 0.1% K_2_HPO_4_, 0.02% MgSO_4_•7 H_2_O and 1.0% Na_2_CO_3_ (all concentrations are given in w/v in distilled water) [16]. The three‐stage fermentation was performed on three different scales (without pH control), which are compared in Table 2. The first 10 mL bioreactor was inoculated with a loop of freshly grown bacterial cells from a 2‐day‐old Petri dish. Between each stage, our aim was to maintain an inoculation ratio of 10 v/v%; therefore, from the first bioreactor, 10 mL of fermentation broth was transferred into a working volume of 100 mL in a B. Braun Biostat Q bioreactor. During the second stage, 80 mL of broth was transferred into 720 mL of Horikoshi II medium in a 1 L B. Braun Biostat Q fermenter. To sustain aerobicity, a special cap, which includes a membrane for filtering the air, was installed on the Biosan RTS‐1C Personal Bioreactor. During the second and third stages, an aeration rate of 0.2 L/min was applied.

**TABLE 2:** Comparison between scales of production

Stage	Bioreactor type	Working volume [mL]	Total volume [mL]	Agitation speed [rpm]	Temperature [°C]	Inoculation ratio [v/v%]
1.	Biosan RTS‐1C Personal Bioreactor	10	50	300	37	N/D
2.	B. Braun Biostat Q	100	300	300	37	10
3.	B. Braun Biostat Q	800	1000	300	37	10

According to our previous results, for the purpose of producing higher enzyme activity, a semi‐continuous fermentation process is favorable when compared to batch and fed‐batch fermentations [12]. However, a three‐stage fermentation can be considered as a semi‐continuous fermentation whereby the working volume increases. The aim was to reduce the long lag phase by starting the third stage of the fermentation.

### 2.3 Biomass analysis

The bacterial growth was monitored by optical density (OD) measurements at 600 nm by a Pharmacia LKB Ultrospec Plus Spectrophotometer. 1.5 mL of broth was transferred into a labelled Eppendorf tube, before being centrifuged at 12,000 rpm for 6 min (Seisystem Bio). The supernatant of this centrifuged sample was the reference (blank) and was diluted to the same extent as the cell‐containing sample.

### 2.4 Enzyme activity measurement

The cell‐containing samples were centrifuged (Seisystem Bio) at 12,000 rpm for 6 min. The cell‐free supernatant was used for further analysis. The measurement of the extracellular CGTase activity was adapted from Kaneko [17] with small modifications. The β‐CGTase activity was measured at 550 nm. During the activity measurements, β‐cyclodextrin was formed by the CGTase from water‐soluble starch which resulted in a colorless complex with purple phenolphthalein. Therefore, the absorbance decrement was measured as follows: 15 mL reaction tubes containing 4.5 mL 50 mM Tris‐HCl buffer (pH = 9) in 1% w/v water‐soluble starch and 0.5 mL of cell‐free supernatant were mixed by a vortex mixer, before being incubated at 40°C in a water bath. Four 0.5 mL samples were taken after 0, 10, 20, and 30 min. These samples were boiled for 5 min to inactivate the enzyme before being transferred into cuvettes containing phenolphthalein solution (1.2 mL 0.06 mM phenolphthalein in a 0.5 M Na_2_CO_3_ solution). The four absorbance values were plotted against time and the gradient (mmol/min) converted into enzyme activity in the form of unit/mL supernatant. One unit of CGTase activity was defined as the amount of enzyme capable of producing 1 μmol β‐CD per min.

### 2.5 Plant processing

*Stevia rebaudiana* plants were kindly donated by Golmitz Kertészet (Golmitz & Golmitz Kft., Hungary, a gardening company). These plants were cultivated in a 1.2 m x 0.6 m x 0.8 m wooden box lit for 16 h (1455 lux) per day at ambient temperature and humidity (25 ± 2°C and 30 ± 10%, respectively). The plants were harvested once they had grown to a height of 50 cm: the leaves were collected separately and lyophilized by a Martin Christ Alpha 2–4 LSC freeze dryer. Once lyophilized, the leaves were stored before later being extracted.

*Stevia rebaudiana* (Bertoni) plants cultivated outside were grown and harvested (during the summer of 2012) from an outdoor plantation in Orosháza, Hungary. The leaves were dried in a room ventilated by air at 25°C for 2 weeks before being stored at room temperature in plastic bags to yield a raw material of uniform quality.

*Stevia rebaudiana* plants were extracted by pressurized hot water extraction according to Németh and Jánosi [18]. Hundred grams of preserved plant extract was mixed with 500 mL of distilled water before being autoclaved at 121°C for 20 mins at 2 bars. Then this mixture was pressed in a small 2 L wine press to separate the plant residue from the extract. This extract containing the steviol glycosides was used in the bioconversions.

### 2.6 Enzymatic bioconversion

A face centered central composite design was created to increase the rebaudioside A content of the plant extract, where the factors were pH (3 – 6 – 9), temperature (15 – 30 – 45°C) and concentration of the glycosyl donor (5 – 15 – 25 g/L). Following our previous research, sucrose was chosen as the glycosyl donor (Figure 1) [4]. The reaction mixture was comprised of 3 mL of cell‐free supernatant, 3 mL of *Stevia rebaudiana* extract and 4 mL of 0.1 M citrate buffer solution containing the glycosyl donor. After mixing the previous components in a 15 mL test tube, 1 mL of the starting sample was extracted before being boiled for 5 min to inactivate the enzyme, then frozen for high‐performance liquid chromatography (HPLC) analysis. The reaction time of the bioconversion was 16 h, at the end of which, 1 mL of the final sample was extracted and treated as the starting sample.

(1) $$Stevioside\;conversion\left(\%\right)=\frac{C_{St}\left(start\right)-C_{St}\left(end\right)}{C_{St}\left(start\right)}*100$$

(2) $$Rebaudioside\;A\;yield\left(-\right)=\frac{C_{P}-C_{P0}}{C_{S0}}$$

(3) $$\begin{array}{ccc} & & Rebaudioside\;A\;conversion\left(\%\right)\\ & & \;=\frac{C_{RA}\left(end\right)-C_{RA}\left(\mathrm{start}\right)}{C_{RA}\left(start\right)}*100 \end{array}$$

In Equation 1, *C*_St_(start) denotes the initial stevioside concentration in the reaction solution and *C*_St_(end) represents the measured stevioside concentration at the end of the reaction. The stevioside and rebaudioside A concentrations were determined using HPLC with a standard calibration curve. Equation 2 shows the calculation of the rebaudioside A yield. In Equation 2, *C*_p_ stands for the rebaudioside A concentration at the end of the bioconversion, *C*_p0_ refers to the initial reb A concentration and *C*_S0_ is the initial stevioside concentration. After the experiments, the rebaudioside A yield was calculated by applying the most promising bioconversion settings. Equation 3 represents the calculation of the reb A conversion, which values are presented in Table 3. In Equation 3, *C*_RA_(start) is the initial concentration of reb A, and *C*_RA_(end) is the reb A concentration after the bioconversion.

### 2.7 HPLC analysis of steviol glycosides

The stevioside and rebaudioside A concentrations in the plant extract and samples were determined by Waters HPLC. The parts of the equipment were the following: a Waters 717 plus autosampler, Waters 1515 isocratic pump and Waters 2487 UV detector. An Agilent ZORBAX carbohydrate analysis column (5 μm, 4.6 × 150 mm) was carried out by a Waters guard column with a mobile phase of acetonitrile and 0.05 M KH_2_PO_4_ buffer solution at a flowrate of 1 mL/min. The detected wavelength was 204 nm and the temperature applied was 25°C. The concentrations of the stevioside and rebaudioside A were calculated by Equation 4 (*R* ^2 ^= 0.9906) and 5 (*R* ^2 ^= 0.9906), respectively.

(4) $$Stevioside\left[g/L\right]=5*10^{-7}*Peak\;Area$$

(5) $$Rebaudioside\;A\left[g/L\right]=2*10^{-7}*Peak\;Area$$

### 2.8 Statistical analysis

Statistica 13.5 (StatSoft, Inc., Tulsa, USA) software was applied for statistical analysis and data visualization. A face centered central composite design was built to investigate the bioconversion of stevioside to rebaudioside A. All the factors were investigated at three levels: temperature (15 – 30 – 45°C), pH (3 – 6 – 9) and sucrose concentration (5 – 15 – 25 g/L).

Equation 6 represents the calculation of relative standard error (RSE), where *standard error* is the standard deviation of the samples’ mean (center points), and *estimate* is the mean of the sample. Relative standard error was applied for estimating the variance of the results in the statistical design's experiment (Table 3).

(6) $$Relative\;standard\;error=\frac{standard\;error}{estimate}*100$$

## 3 RESULTS AND DISCUSSION

### 3.1 CGTase enzyme fermentation

**FIGURE 2:**
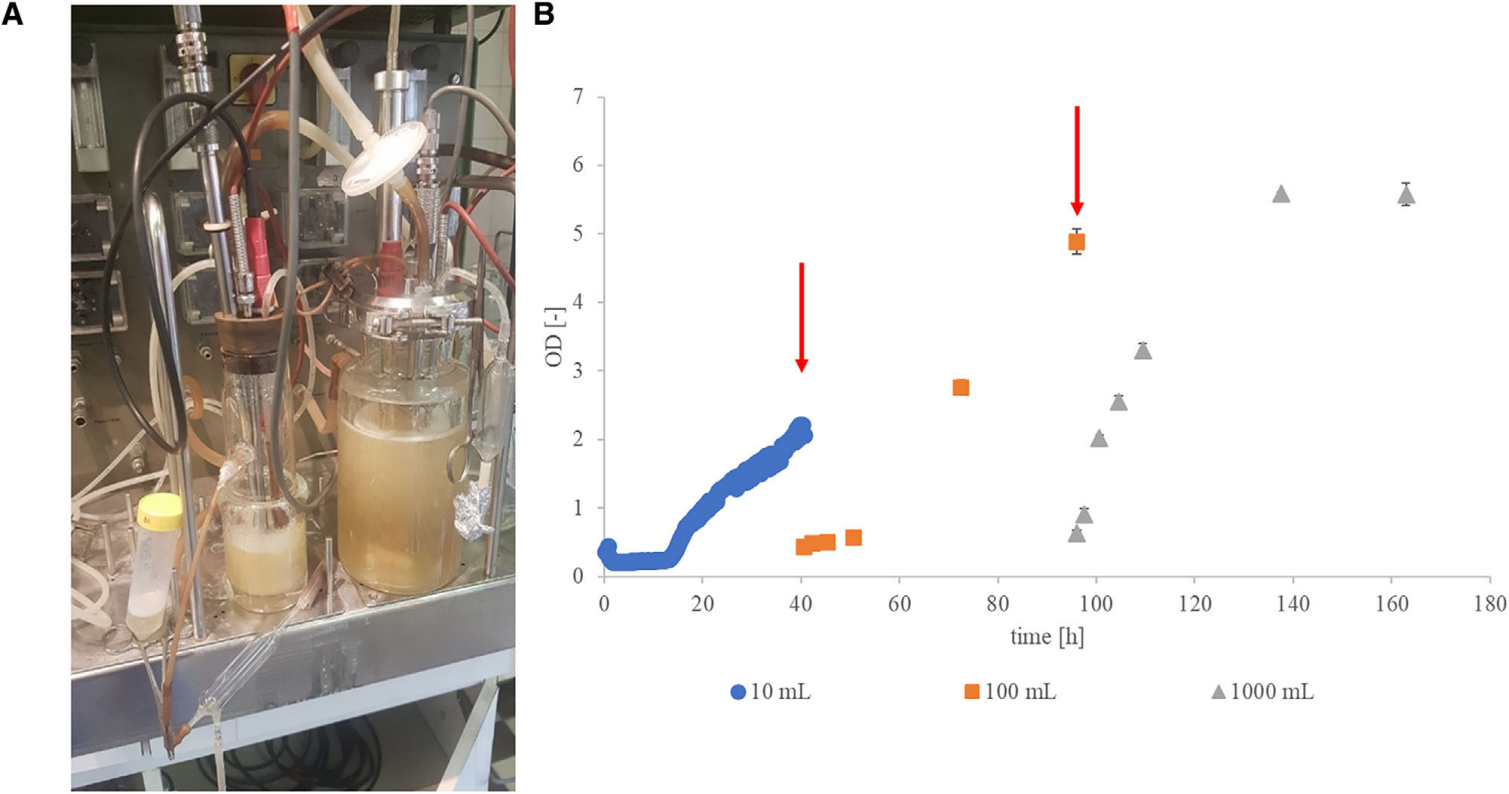
(A) Applied bioreactors during CGTase production (left: Biosan RTS‐1C, middle: B. Braun Biostat Q 100 mL, right: B. Braun Biostat Q 1 L). (B) Cell growth during the scale‐up (red arrows indicate the end of the inoculum‐producing stages; OD, optical density)

During the three‐stage fermentation, the first two stages yielded the inoculum required for the 3rd stage, that is, the enzyme producing one on a 1 L scale. The applied bioreactors used to produce the enzyme are presented in Figure 2A. The aim of this fermentation technique was to reduce the lag phase during the 3rd stage. By analyzing Figure 2, it can be seen how the lag phase of the bacteria is shortened by extending the timescale. In the case of the Biosan RTS‐1C, a 12‐h‐long lag phase can be observed. This period of adaptation was reduced to 4 h in the 2nd stage. Finally, in the 3rd stage, this lag phase was completely eliminated. Optical density indicated with almost constant values the turning of the culture into declining phase after which both fermentation and spore formation of the bacteria ended. By the end of the 100 mL and 1000 mL stages, the biomass concentration reached 4.78 and 5.59 g/L, respectively.

There are various types of fermentation techniques for the production of CGTase enzyme. However, the most commonly applied technique is the batch fermentation typically in shaking flasks [19]. Additionally, there are a few studies where researchers deal with the production of CGTase enzyme in bioreactors as semi‐continuous systems. Costa et al. (2015) and Czinkóczky and Németh (2019) fermented CGTase enzymes, with semi‐continues technique by *Bacillus circulans* DF 9R and *Bacillus licheniformis* DSM13, respectively [12, 20]. Continuous fermentation has many advantages contrary to the batch or repeated batch fermentations, likewise higher productivity and less downtime. Abdel‐Naby et al. (2011) compared the productivity of free cell and immobilized cell systems in batch, repeated batch and continuous systems in small scale with *Bacillus cereus* NRC7 cells. Using immobilized cells they reached a 10‐fold increasement in the continuous system comparing to the batch one [21]. For large scale production the increased productivity is essential to feasible bio‐process. Thus, the batch‐wise fermentation seems to be a viable operation for CGTase production.

In our experiments by the end of the enzyme‐producing fermentation, the final CGTase activity was 2.58 ± 0.37 U/mL. This cell‐free supernatant was applied during the enzymatic bioconversions. *Bacillus licheniformis* DSM13 can be regarded as an exceptional CGTase‐producing wild‐type strain. Among *Bacilli*, several potential CGTase‐producing strains exist, e.g. *Bacillus lehensis* (0.45 U/mL) [22], *Bacillus licheniformis* (isolated from Sao Paolo, 0.162 U/mL) [23], *Bacillus circulans* DF 9R (1.47 U/mL) [20] and *Bacillus licheniformis* DSM13 (2.40 U/mL) [12].

### 3.2 Enzymatic bioconversion of steviol glycosides

Transglycosylation of stevioside to rebaudioside A was studied with fermented CGTase from *Bacillus licheniformis* DSM13 and with sucrose as glycosyl donor. Different sucrose concentrations, temperature and pH levels were tested in order to increase the reb A content in the *S. rebaudiana* extract to gain a sweeter, less bitter bioproduct.

The initial concentrations were similar in the case of both plant extracts. The initial stevioside and rebaudioside A concentrations are presented in Table 4, while the raw data of the experimental results are summarized in Table 3.

**TABLE 3:** Average steviol glycoside content of the dried and freeze‐dried plant extracts

	Dried plant extract	Freeze‐dried plant extract
Stevioside [g/L]	9.32 ± 0.96	8.44 ± 0.68
Rebaudioside A [g/L]	1.52 ± 0.38	1.33 ± 0.12

**TABLE 4:** Final rebaudioside A concentrations with relative standard errors after the bioconversions and rebaudioside A conversions

			Dried plant		Lyophilized plant	
pH [‐]	T [°C]	Sucrose [g/L]	reb A [g/L] ± RSE	reb A conversion [%]	reb A [g/L] ± RSE	reb A conversion [%]
3	15	5	6.40 ± 0.68	412.04	7.22 ± 0.59	391.18
9	15	5	0.82 ± 0.09	‐34.59	1.47 ± 0.12	‐0.09
3	45	5	2.41 ± 0.26	92.74	2.24 ± 0.18	52.05
9	45	5	1.89 ± 0.20	51.02	1.72 ± 0.14	17.34
3	15	25	4.77 ± 0.51	281.36	2.31 ± 0.19	57.02
9	15	25	1.87 ± 0.20	49.57	0.92 ± 0.08	‐37.56
3	45	25	1.52 ± 0.16	21.79	2.12 ± 0.17	44.33
9	45	25	1.95 ± 0.21	55.81	1.86 ± 0.15	26.30
6	30	15	3.82 ± 0.41	205.96	4.34 ± 0.36	195.24
6	30	15	5.08 ± 0.54	306.54	3.42 ± 0.28	132.65
6	30	15	5.54 ± 0.59	343.11	3.42 ± 0.28	132.65
3	30	15	2.13 ± 0.23	70.68	2.61 ± 0.21	77.51
9	30	15	2.02 ± 0.22	61.82	2.00 ± 0.16	36.06
6	15	15	3.16 ± 0.34	152.91	2.06 ± 0.17	40.47
6	45	15	1.42 ± 0.15	13.33	2.88 ± 0.24	95.69
6	30	5	0.81 ± 0.09	‐34.97	3.00 ± 0.025	104.33
6	30	25	1.43 ± 0.15	14.75	3.49 ± 0.29	137.60

After the experiments, the results were analyzed using Statistica 13.5 software. First, the distribution of the data was checked. The residual plots in both cases were very similar. The histogram, the normal probability plot and predicted versus residual values indicate that the data is of normal distribution and its variance is constant.

#### 3.2.1 Enzymatic bioconversion using dried plant extract

**FIGURE 3:**
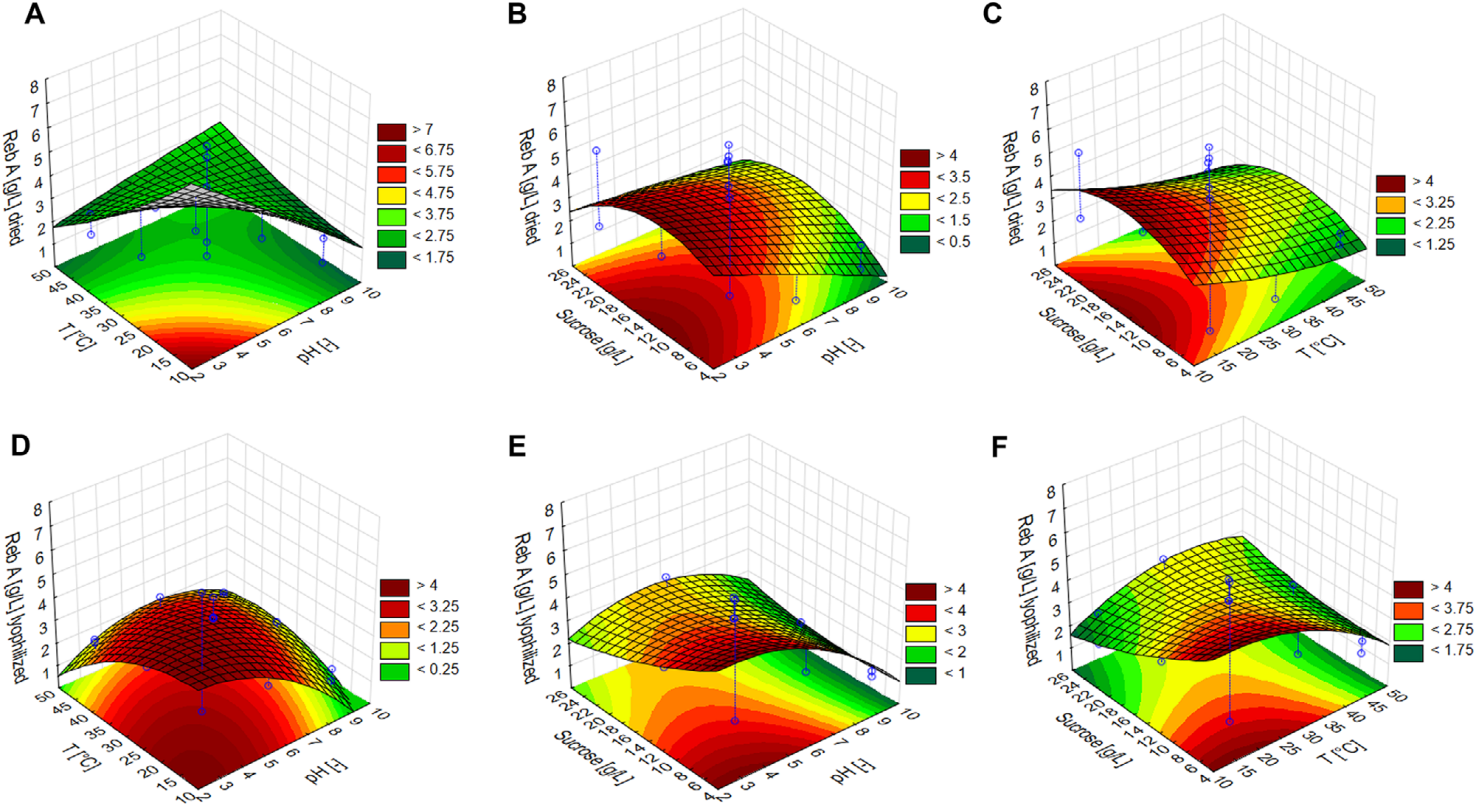
(A‐C) Surface plots of the achieved rebaudioside A concentrations using dried *Stevia rebaudiana* extract and (D‐F) surface plots of the achieved rebaudioside A concentrations using lyophilized *Stevia rebaudiana* extract

The surface plots after the statistical analysis are presented in Figure 3A‐C. From Figure 3, it can be seen, that a lower temperature (approximately 10–20°C) is favorable for achieving a higher final concentration of rebaudioside A. Moreover, although a strongly acidic pH also facilitates the production of a higher reb A content at the end of the bioconversion, varying the sucrose concentration had no effect.

#### 3.2.2 Enzymatic bioconversion using lyophilized plant extract

The surface plots created after the statistical analysis of the lyophilized plant extracts are shown in Figure 3D‐F. It can be observed that higher concentrations of rebaudioside A were achieved at lower levels of the investigated variables i.e. temperature, pH, and sucrose concentration.

By analyzing the trends as a result of varying the pH, while ignoring the one outlying data point at a reb A concentration of 7 g/L, the centrum points (pH = 6, T = 30°C, sucrose = 15 g/L) seem to be more reliable in terms of enzymatic bioconversion. The pH had a significant effect (*p* = 0.03374 at a 95% confidence level) on rebaudioside A enrichment.

### 3.3 Discussion of enzymatic bioconversion results

The conventional method of the enzymatic bioconversion of *S. rebaudiana* extract using sucrose as a glycosyl donor showed that at almost all settings the final rebaudioside A concentration was increased. Therefore, the fermented CGTase enzyme extracted from *Bacillus licheniformis* DSM13 is an excellent candidate for a subsequent scale‐up and industrial production of reb A enriched *S. rebaudiana* extract. Similarly to our previous research when sucrose was applied as a glycosyl donor but β‐1,3‐glucanase extracted from *Trichoderma longibrachiatum* as a catalyst, the same conditions are favorable for the enrichment of rebaudioside A during the bioconversion, namely acidic pH and low temperature [4].

In comparison with our results, Li et al. (2012) applied a commercial CGTase enzyme (Toruzyme 3.0 L) in a conventional bioconversion and achieved a 77.11% stevioside conversion to subdue the bitter taste of stevioside. This finding is comparable with our highest stevioside conversion (83 ± 5%) as well as our highest reb A concentrations. Stevioside conversions of between 60% and 70% are achieved using non‐conventional microwave‐assisted bioconversions [24, 25]. Rebaudioside A yields at a pH of 3, temperature of 15°C and sucrose concentration of 5 g/L were 0.331 and 0.665 for dried and lyophilized plant extracts, respectively. Although the initial reb A concentrations were similar for both raw materials tested (the average initial concentrations over 17 runs were 2.67 ± 1.32 and 2.55 ± 0.66 g/L for dried and freeze‐dried *S. rebaudiana* extracts, respectively), the significant difference in yields can be explained by the fact that freshly and quickly freeze‐dried leaves contained less compounds that inhibited decomposition in comparison with plant biomass that was dried slowly at room temperature.

The same enzymatic bioconversion was not published until yet using *S. rebaudiana* extract with CGTase enzyme and saccharose as glucosyl donor.

Regarding optimum searching for conventional enzymatic treatment of *stevia plant‐biomass* for enhancing reb A content, Adari et al (2016) observed a pH optimum of 4.6 for *Trichoderma viridae* cellulase enzyme on *S. rebaudiana* leaves [26]. Li et al (2013) investigated *conventional CGTase* enzymatic bioconversion on pure stevioside and observed a pH optimum of 5–6 and temperature optimum of 60°C applying starch as glycosyl donor [28].

## 4 CONCLUDING REMARKS

Rebaudioside A is a natural diterpene glycoside which can be 300 times sweeter than sucrose, moreover, is of nutritional value regarding human nutrition unlike stevioside. Since only a handful of studies on the enzymatic bioconversion of stevioside extracted from *S. rebaudiana* leaf extracts into reb A are found in the literature, the enzymatic enrichment of *S. rebaudiana* leaf extract with reb A was sought.

During our research, on average, the rebaudioside A content doubled by the end of the enzymatic bioconversion catalyzed by fermented CGTase enzyme; however, the highest increase was fivefold. From these results, it can be concluded that the cyclodextrin glycosyltransferase from *Bacillus licheniformis* DSM13 is a suitable enzyme with regard to producing rebaudioside A from stevioside. No significant differences between the stevioside and rebaudioside A concentrations of the two plant‐based biomasses used as raw materials were observed. On the contrary, by analyzing the highest yields of rebaudioside A, a twofold difference between the dried and lyophilized plant extract was recorded. From our experiments, it can be said that the pressurized hot water extraction of the plant *Stevia rebaudiana* is a very suitable raw material in the field of enzymatic bioconversion.

In the near future, other *Bacillus* species will be screened for suitable CGTase enzymes and the fermented enzymatic activity will be evaluated through the bioconversions of pure stevioside solutions and *Stevia rebaudiana* leaf extracts.

## CONFLICT OF INTEREST

The authors have declared no conflict of interest.

## Data Availability

The data that support the findings of this study are available from the corresponding author upon reasonable request.
